# Integrating Inertial Sensors to Assess Physical Performance and In-Match Demands for the International Selection of Cerebral Palsy Football Players

**DOI:** 10.3390/s25185787

**Published:** 2025-09-17

**Authors:** Juan F. Maggiolo, Raúl Reina, Manuel Moya-Ramón, Iván Peña-González

**Affiliations:** 1 Department of Sports Sciences, Sports Research Centre, Miguel Hernández University of Elche, 03202 Elche, Spain; fran.maggiolo@goumh.umh.es (J.F.M.); rreina@umh.es (R.R.); mmoya@umh.es (M.M.-R.); 2 Spanish Federation of Sports for People with Cerebral Palsy and Acquired Brain Injury (FEDPC), 28011 Madrid, Spain

**Keywords:** wearable sensors, soccer, talent identification, physical performance, para-sport, brain impairment

## Abstract

**Highlights:**

**What are the main findings?**

- International CP football players show superior sprinting, change-of-direction, and dribbling performance in field tests compared to national-level players, particularly in FT1 and FT2 sport classes.
- In FT2 players, high-intensity running during matches, measured by inertial sensors, was the most accurate predictor of international selection status.

**What is the implication of the main finding?**

- Physical performance tests and wearable sensor data can objectively differentiate elite CP football players, providing evidence-based tools for talent identification.
- Integrating these assessments into national selection strategies may enhance the accuracy and fairness of international team recruitment in para-football.

**Abstract:**

This study analyzed the physical performance (via field tests) and in-match physical responses (via wearable inertial sensors) of national and international cerebral palsy (CP) football players competing in Spain’s First Division. A total of 80 players (FT1: n = 22; FT2: n = 48; FT3: n = 10) completed sprinting, change of direction, and dribbling tests. In-match data from 74 players were collected across 56 official matches. Players were classified as “international” (candidates for the national team) or “national” (non-candidates). Statistical analyses identified performance differences and predictors of international selection using multiple discriminant analysis. International players outperformed national ones in sprinting, agility, and dribbling, especially in FT1 and FT2 classes (*p* < 0.05; large effect sizes). In-match data (analyzed for FT2 only) showed that international players covered more distance at all intensities and executed more high-intensity actions (e.g., maximal velocity, ball contacts). High-intensity running was the strongest predictor of international status (74.5%, Wilks’ λ = 0.86, *p* = 0.01). Change of direction and dribbling were key discriminators in FT1 and FT2, while no clear predictor emerged in FT3. These findings support the use of physical tests and wearable technology for evidence-based talent identification and selection in CP football.

## 1. Introduction

Cerebral palsy (CP) football is an internationally recognized 7-a-side football modality played by people with CP or acquired brain injury and governed by the International Federation of Cerebral Palsy Football (IFCPF). It is a high-intensity team para-sport characterized by explosive actions such as sprints, changes of direction, and accelerations, interspersed with periods of low-intensity activities or recoveries. The assessment of physical performance in CP football has become an increasingly relevant area of research, given its impact on talent identification, players sport classification, or training planning [1].

Performance in CP football is influenced by multiple factors, including sports classification, which categorizes players into three sport classes (i.e., FT1, FT2, and FT3) based on how the three eligible impairments of spastic hypertonia, motor ataxia or dyskinesia (including body region affected and severity of the impairment) constraint the performance of fundamental football skills (i.e., activity limitation). The recent incorporation of wearable sensing technologies, such as inertial measurement units (IMUs), has enabled accurate and non-invasive monitoring of athletes’ physical requirements [2]. In CP football, where traditional performance metrics are often limited by motor impairments, the use of such technology provides an objective and reproducible way to evaluate functional performance during match play. However, research applying IMUs for talent identification in para-sports remains limited.

Previous studies have demonstrated that FT1 players experience greater limitations in high-intensity actions, while FT3 players, whose impairments have a lesser impact on movement, tend to exhibit superior performance in speed and change-of-direction abilities [3]. These performance differences can be explained by the functional impact of the underlying impairments. For instance, athletes in the FT1 class typically present with more severe spastic hypertonia, ataxia, or dyskinesia, which limit rapid coordination, balance, and force production, thereby restricting their ability to execute explosive football-specific actions such as sprinting, sharp directional changes, and powerful shooting. Due to these limitations, FT1 players often assume the goalkeeper role within their teams, as the high impact of their impairment constrains their capacity to perform outfield technical and physical actions effectively [4]. In contrast, FT2 players generally present with moderate impairments that still affect coordination and stability, but allow for greater execution of technical actions such as ball control, passing, and dribbling under pressure. Finally, FT3 players, whose impairments are milder, are better able to maintain postural control and coordination, facilitating more effective performance in speed-related actions, changes of direction, and technical skills such as accurate passing and finishing. Research by Yanci et al. (2018) confirmed that players with less severe impairments (i.e., former FT8 sport class or mild impairment) covered greater distances at high-intensity speeds (>13 km·h^−1^) and performed more accelerations, decelerations, and changes of direction than those belonging to the FT5/6 (moderate impairment of spastic diplegia or ataxia/athetosis, respectively) and FT7 (moderate spastic hemiplegia) sport classes [5]. Similarly, Gamonales et al. (2022) conducted a full-season analysis of CP football and found that FT3 players consistently covered more total distance, sprinted at higher speeds, and executed a greater number of moderate and high-intensity accelerations compared to FT1 and FT2 players [6]. Further evidence supporting these distinctions highlighted that FT1 players demonstrate lower proficiency in activities requiring rapid coordination, balance, and force production, which directly affects their ability to perform explosive movements such as sprinting and sharp directional changes [7]. Likewise, Yanci et al. (2021) emphasized the role of dribbling and change-of-direction ability skills in differentiating between sport classes, suggesting that these physical capabilities are crucial for competitive success [3]. Moreover, external workload analysis of CP football matches indicated that FT3 players not only performed more high-intensity movements but also engaged in more game-related actions involving technical execution, such as passing or shooting [8]. These findings underscore the competitive advantage that FT3 players possess, given their ability to maintain high-intensity performance throughout the match. The implications of these findings are significant for talent identification and training program development, as they highlight the necessity of tailoring assessment, player’s selection and conditioning programs to the specific physical demands of each sport class. In addition, it should be considered that CP football regulations limit the participation of FT3 players to only one per team on the field of play, while mandating that at least one FT1 player throughout the entire match [9].

However, the literature on talent identification and selection processes in this sport remains scarce. The selection of players for national and international CP football teams has traditionally been a subjective process based on coaches’ experience or players’ availability/recruitment. However, in recent years, scientific research has introduced various performance assessment tests for this population [1]. These previous studies have demonstrated the validity, reliability, and utility of different field tests in effectively evaluating the physical performance of CP football players [10]. Among these, acceleration and linear speed (5 to 30 m sprint), change-of-direction, and dribbling tests have shown a strong ability to differentiate physical performance levels among CP football players [10]. Peña-González et al. (2021) were the first to identify which of these tests might be most relevant to the talent identification and selection process for international CP football players, highlighting change-of-direction and dribbling tests as the most relevant, although their importance may vary depending on the sport class [11]. Moreover, recent findings indicate that players’ performance in these field tests is correlated with their physical demands in competition, such as moderate- and high-intensity accelerations and running efforts [12]. This suggests that players who achieve higher scores in field tests may also exhibit greater physical demands during matches, which could serve as a valuable tool for talent identification and selection of CP footballers.

However, while previous studies have provided valuable insights into differences between sport classes and have highlighted the role of specific physical capabilities in CP football, little is known about whether these findings also extend to differences across competitive levels. In particular, there is a lack of evidence examining how the physical performance and in-match demands of national players compare with those of athletes competing at the international level. Such a comparison is essential to better understand the factors that underpin progression to elite competition, to refine talent identification strategies, and to support evidence-based player selection for international events. Therefore, the aim of the present study was to analyze the anthropometric and physical performance (through field tests) and in-match physical requirements (using inertial devices during competition) of national and international players during matches in the First Division of the National CP Football League (NCPFL), organized by the Spanish Federation of Sports for Individuals with CP and acquired brain injury (FEDPC). It was hypothesized that international players will achieve higher values in both physical performance and physical requirements recorded during competition. However, to the best of the authors’ knowledge, no prior scientific evidence allows for the formulation of a hypothesis regarding which variables will best differentiate between national- and international-level players. Consequently, further investigation is needed to determine the key variables to be considered in the talent identification and selection process for international CP football players.

## 2. Materials and Methods

### 2.1. Design

This is a cross-sectional study conducted over an entire season of the First Division of NCPFL for individuals with CP and acquired brain injury, organized by the FEDPC. Throughout the seven regular-season matchdays and the two playoff matchdays, physical performance tests were conducted (randomly assigned to each team on a matchday), and the physical demands of the competition were recorded in all matches (n = 56 games) using a wearable global positioning system (GPS) device.

### 2.2. Participants

This study aimed to assess 100% of the sample comprising the First Division of the NCPFL. Out of the 93 players with an active license in the league (January−May 2024), the physical performance (through field tests) of 80 players was evaluated. The reasons for not assessing the remaining players were: being a goalkeeper (excluded from the study), not attending the matchday when their team’s evaluation was conducted, or being injured or unwell. Ultimately, the physical performance of 80 male CP football players was assessed (FT1: n = 22, body height = 1.75 ± 0.09 m, body weight = 66.62 ± 9.48 kg; FT2: n = 48, body height = 1.76 ± 0.06 m, body weight = 62.27 ± 8.19 kg; FT3: n = 10, body height = 1.73 ± 0.10 m, body weight = 65.21 ± 6.75 kg). To assess the physical demands during competition, the primary inclusion criterion was to participate in at least one match as a starting outfield player. Of the 93 players with an active license in the 2024 league, 74 met this selection criterion (FT1 = 10; FT2 = 55; FT3 = 9). For both comparisons (physical performance and in-match physical demands), participants were categorized as “international” players (those included in the national team monitoring group as candidates for selection for the IFCPF World Cup, held in November 2024) or “national” players (those outside the national team monitoring list). All international players had been called up for at least three training camps with the national team and had participated in at least one international tournament. All participants were informed of the study’s objectives and provided written informed consent in accordance with the Declaration of Helsinki. This study was approved by the Ethics Committee of the researchers’ affiliated university (REF: ADH.DES.IPG.JFM.2).

### 2.3. Procedures

The 2024 FEDPC NCPFL consisted of seven regular-season matchdays followed by two final-phase matchdays. During the regular-season matchdays, teams were randomly selected to undergo their physical performance assessment prior to their scheduled match on that matchday. First, players’ height and weight were measured using a portable stadiometer (SECA Ltd., Hamburg, Germany, ±0.1 cm) and a digital scale (Tanita Bc 601 Ltd., Tokyo, Japan, ±0.1 kg), respectively.

#### 2.3.1. Physical Performance Assessment

A standardized warm-up protocol was conducted, consisting of 3 min of low-intensity running, 5 min of joint mobility exercises and dynamic stretching, followed by 3 min of high-intensity actions, including jumps, accelerations, and changes of direction [13]. Following the warm-up, a battery of field tests was performed to assess players’ linear velocity, change of direction ability, and dribbling skills. All tests were conducted on an artificial turf football field, with players wearing their specific football boots. Coaches and researchers encouraged players throughout the tests to ensure maximum effort. Each test was performed twice, with 2 min of rest between repetitions, and the best result for each was recorded for further analysis. During all tests, players started from an upright position, with their feet positioned 30 cm behind the starting line. Timing for each test was recorded using a set of photocell timing gates (Witty System, Microgate, Bolzano, Italy).

Linear Velocity. To assess acceleration and linear velocity, a 30 m sprint test was performed, with split times recorded at 5, 10, 15, 20, and 30 m.

Change of Direction. To assess players’ change of direction ability, the Modified Agility Test (MAT) [14] was used, applying the version by Peña-González et al. (2021), in which players always run in a forward direction and are not required to touch the cones before changing direction [11].

Dribbling Test. To assess players’ ability to run and change direction while dribbling the ball, the same MAT structure was used, but with players required to dribble the ball between the cones instead of changing direction without the ball [11]. To determine dribbling ability, the researchers subtracted the time recorded in the change of direction test from the time recorded in the dribbling test. The resulting value, termed “Dribbling Ability” (Drib-Ab), reflects a player’s proficiency in handling the ball while running, with lower values indicating a higher skill level, independent of their acceleration or change of direction ability.

#### 2.3.2. In-Match Physical Requirements Assessment

Players’ physical requirements during competition were recorded using wearable devices (OLIs; Oliver IMU®, Barcelona, Spain). The OLI system integrates a GPS module sampling at 10 Hz and an inertial measurement unit (IMU; accelerometer and gyroscope) sampling at 50 Hz, which are standard frequencies in tracking devices commonly used in football. The validity of OLI devices has been confirmed in football and futsal, showing high agreement with other commercial systems [15,16]. Moreover, these devices have previously been applied in CP football, where they demonstrated discriminant validity by identifying physical differences between players of different competitive levels [17,18]. To ensure proper calibration, all devices were initialized in a static position during start-up and were always fitted by the same researcher following standardized wearing specifications. Unlike other tracking systems, the OLI is positioned on the posterior side of the player’s dominant leg, which enables not only the quantification of locomotor demands but also the measurement of both the quantity and velocity of ball contacts. The device does not transmit data in real time; instead, data are stored locally during the session and subsequently synchronized via Bluetooth 5 with the OLIVER^®^ software, which processes and visualizes the information. No abnormal or unrealistic values were observed. The variables extracted from the OLI device included the following:

Maximal velocity (km·h^−1^): the highest recorded movement velocity achieved by the player during the match.

- Total distance (m): the total meters covered by the player during the match.
- Walking (m): distance covered while walking (<6 km·h^−1^).
- LI running (m): distance covered at low-intensity running (6–12 km·h^−1^).
- MI running (m): distance covered at moderate-intensity running (>12–18 km·h^−1^).
- HI running (m): distance covered at high-intensity running (>18 km·h^−1^).
- Ball contacts (n): total number of contacts with the ball.
- LI ball contacts (n): number of low-intensity (ankle velocity <11 m·s^−1^ but acceleration >20 G) contacts with the ball.
- MI ball contacts (n): number of moderate-intensity (ankle velocity 11–15 m·s^−1^) contacts with the ball.
- HI ball contacts (n): number of high-intensity (ankle velocity >15 m·s^−1^) contacts with the ball.
- Striking force (km·h^−1^): maximum velocity reached during a ball strike in the match.
- MI accelerations (m): distance covered with moderate-intensity accelerations (>2 to 3 m·s^−2^).
- HI accelerations (m): distance covered with high-intensity accelerations (>3 m·s^−2^).
- MI decelerations (m): distance covered with moderate-intensity decelerations (<−2 to −3 m·s^−2^).
- HI decelerations (m): distance covered with high-intensity decelerations (<−3 m·s^−2^).

All variables, except for maximal velocity and maximum striking force, were normalized to each player’s playing time to obtain “per minute” values. This normalization aimed to prevent bias due to differences in playing time across players.

### 2.4. Statistical Analysis

The sample was divided for analysis based on sport class, due to the clear functional differences among them. A Shapiro–Wilk test was conducted to verify whether the variables in the national and international player subgroups followed a normal distribution. To assess differences in physical performance tests between national and international players, a Mann–Whitney U test was performed due to the small and unbalanced sample size of international players (FT1: 18 national vs. 4 international players; FT2: 31 vs. 17; FT3: 7 vs. 3) and the non-normal distribution of the data (W = 0.84–0.90, *p* < 0.050). Physical performance variables were further analyzed using a stepwise multiple discriminant analysis to identify which physical performance test variables best predicted a player’s international selection [19]. For in-match physical requirement variables, statistical analyses could not be performed for national vs. international comparisons in FT1 and FT3 due to small sample sizes (FT1: 9 vs. 1; FT3: 7 vs. 2). However, for FT2 (n = 38 vs. 17), a Mann–Whitney U test was conducted to compare national and international players, given the small and unbalanced sample size and the non-normal distribution of the variables (W = 0.76–0.94, *p* < 0.050). A stepwise multiple discriminant analysis was performed in the FT2 sample to identify the in-match physical requirement variables that best predicted international selection. The standardized differences or effect sizes (ES) between national and international players, along with their 95% confidence intervals (CI), were expressed as rank-biserial correlation coefficients (r_β_) and interpreted as follows [20,21]: trivial (<0.10); small (0.10–0.29); moderate (0.30–0.49); and large (> 0.50). All data were processed and analyzed using Microsoft Excel (Microsoft, Seattle, WA, USA), JASP software (JASP for Windows, version 0.13, Amsterdam, The Netherlands), and SPSS Statistics (Statistical Package for the Social Sciences, Version 27.0, IBM Corp, Armonk, NY, USA). The level of significance was set at *p* < 0.050.

## 3. Results

### 3.1. Physical Performance

**Figure 1:**
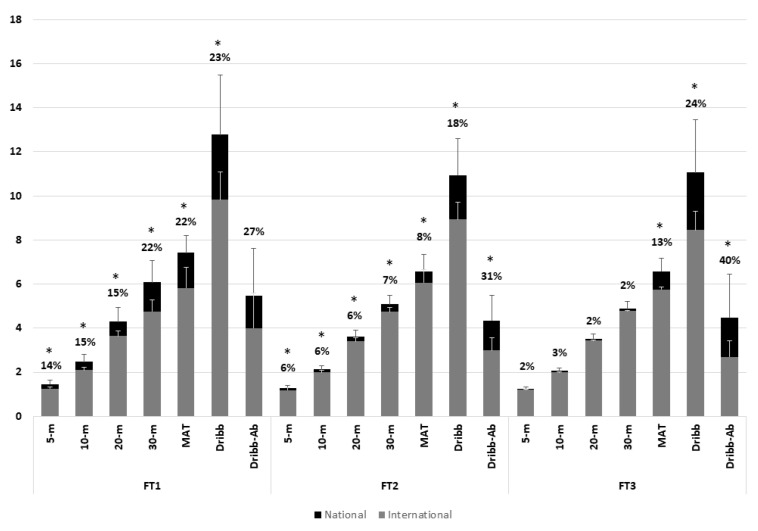
Physical performance difference, in percentage (%), between national and international players in each sport class. * *p* < 0.050.

Descriptive data for national and international players according to their sport class is shown in Table 1. No significant differences were found in body weight or height between national and international players in any sport class. However, the ES indicated small to moderate differences between groups in these variables across sport classes. The percentage differences between national and international players in the physical performance tests are shown in Figure 1. For FT1 class, significant differences and large ES were found between groups for all physical performance tests except to the dribbling ability (moderate ES). For FT2 class, all physical performance tests presented significant differences and large ES between groups, excepting the 5 m sprint that presented a moderate ES. For FT3 class, just the MAT, dribbling test and dribbling ability presented significant differences between subgroups. All the tests had a large ES between subgroups except the 5 m sprint (moderate ES).

Box’s M test confirmed the assumption of equal covariance matrices for each sport class (M = 0.14−1.97, F = 0.12−1.69, *p* > 0.050), ensuring the robustness of the discriminant function analysis. Significant variables for each sport class are presented in Table 2. The model retained MAT as the main relevant predictor in FT1 (eigenvalue = 0.64; canonical correlation = 0.63; explained variance = 100%) and the dribbling test in FT2 (eigenvalue = 0.42; canonical correlation = 0.55; explained variance = 100%), while no prediction model was shown for FT3. These models correctly categorized 100% of national players and 25% of international players in FT1, while in FT2 the models correctly categorized 89.3% of national and 81.3% of international players.

### 3.2. In-Match Physical Requirements

Table 3 presents the differences between national and international players in the FT2 class. International players demonstrated higher maximal velocity (U = 187.00, *p* = 0.013, moderate ES), as well as greater low-intensity, moderate-intensity, high-intensity, and overall running distance (U = 150.00−209.00, *p* = 0.002−0.038, moderate ES), and greater moderate, high-intensity, and overall ball contacts (U = 178.50−205.00, *p* = 0.009−0.032, moderate ES). Although without significant values, striking force, and moderate- and high-intensity accelerations presented moderate ES between national and international players.

Box’s M test confirmed the assumption of equal covariance matrices (M = 0.48, F = 0.46, *p* = 0.496), ensuring the robustness of the discriminant function analysis. Wilks’ Lambda test indicated a significant difference for HI running (λ = 0.859, F = 8.67, *p* = 0.005), confirming its role in distinguishing between groups. Other variables such as maximal velocity (λ = 0.90, F = 5.74), total distance (λ = 0.91, F = 5.17), MI running (λ = 0.88, F = 7.14), LI running (λ = 0.88, F = 7.14), and HI contacts (λ = 0.89, F = 5.96), were significant predictors (*p* < 0.050) of national-international player’s aggrupation. The model retained HI running as the main relevant predictor, with a canonical discriminant function eigenvalue of 0.16, a canonical correlation of 0.38, and an explained variance of 100%. The structure matrix revealed that HI running had the strongest correlation with the discriminant function (r = 1.00), followed by LI running (r = 0.87) and Total distance (r = 0.84), although only HI running was included in the final model. Regarding classification accuracy, the model correctly classified 74.5% of the cases, with 97.4% correct classification for national and 23.5% for international group, reinforcing the discriminative power of the HI running variable.

## 4. Discussion

The present study aimed to analyze the anthropometric and physical performance (through field tests) and in-match physical requirements (using inertial measurement devices) of national and international CP football players competing in the First Division of the Spanish NCPFL. The results confirmed the hypothesis that international players exhibit superior performance in both field tests and match-related physical requirements. MAT and dribbling tests and distance covered at HI running were identified as the main predictable variables to correctly identify a player as national or international player. In mainstream football, anthropometric variables have been identified as a factor that could discriminate between elite and non-elite football players [22]. However, in the present study, no differences in weight and height variables were found between national and international players across any of the sport classes. This suggests that these variables may have less relevance or association with sports performance in CP football compared to mainstream football. For instance, it has been demonstrated that significantly fewer headed goals (from aerial crosses or set plays) occur in CP football, and it has been suggested that the smaller field and goal dimensions, as well as greater limitations in coordination and balance for such actions (e.g., jumping for a header), could be the underlying causes of this phenomenon [4]. The reduced necessity to win aerial duels to score or prevent goals may imply that height is not a crucial criterion for talent identification and selection in CP football. In mainstream football, it has also been suggested that the specific functions of players in different field positions could be a key factor in selecting taller or heavier players for certain positions [23,24]. However, no anthropometric differences have been found between playing positions in CP football [25]. This could be due to a lower degree of positional specialization, given that CP football is played in a 7-a-side format and on a smaller pitch (i.e., 70 × 50 m). Additionally, it may be attributed to a larger talent pool in mainstream football and a smaller disparity in physical performance among players, which could lead to anthropometric factors playing a more significant role in the final selection of a player.

Physical performance, assessed throughout field tests, has been traditionally assessed in mainstream football into talent identification and selection strategies and training programs [26]. Physical tests such as sprint, change of direction or dribbling tests has been shown as discriminant at early ages to detect future professional and non-professional players [27,28]. The findings of this study indicate that international CP football players consistently outperformed national players in linear acceleration and velocity, change-of-direction, and dribbling tests, with large ES in FT1 and FT2 sport classes. This aligns with previous research suggesting that high-intensity actions such as sprinting and agility (with and without ball) are crucial for talent identification in CP football [11]. However, this study revealed greater differences between subgroups than those reported by Peña-González, Sarabia et al. (2021), who found no significant differences in performance between selected and non-selected FT1 players for the national team in jumping, sprinting, change of direction, and dribbling skills [11]. Only dribbling performance was significantly different for FT2 players. The larger sample size compared to that of Peña-González [11], Sarabia et al. (2021) may be the main reason for the differences observed in the present study within these sport classes, as well as the increased development of physical conditioning programs for these para-athletes in recent years [29]. The multiple discriminant analysis used in this study showed the MAT as the most relevant predictor variable in FT1 players, while time in the dribbling test (but not the dribbling ability) was the main predictor variable in FT2. These results reinforce previous studies emphasizing the role of change of direction ability and physical-technical skills (i.e., dribbling) in distinguishing international CP football players [1,11].

For FT3 players, only MAT and dribbling-related tests (but not linear sprints) showed significant differences between national and international players. The percentage differences and ES between groups showed that linear velocity seems to be more important in FT1 to discriminate between national and international players, while the dribbling (test and ability) seem to be the more differentiator factor for FT3, while sprint ability seem not to be as important in this sport class. This may be attributed to the higher functional capabilities of FT3 players, among whom performance differences in this ability are minimal (SD = 0.08 s in the 5 m sprint to 0.31 s in the 30 m sprint). The FT3 player—bearing in mind that only one is allowed on the field at any given time—is the player with the least activity limitations (i.e., the most functionally capable player) and typically assumes a more prominent role in the game. Although there are no qualitative studies available to validate this information, it is plausible that coaches demand more complex skills (e.g., dribbling or tactical ability) from FT3 players due to their greater influence on the game and the relatively small differences in sprint performance among eligible FT3 candidates. The higher importance of dribbling ability in this sport class (higher difference between groups compared to the other sport classes; Figure 1) suggests that technical proficiency could be a decisive factor in differentiating elite FT3 players, rather than pure speed or acceleration, which is in line with previous research [11]. However, the multiple discriminant analysis did not select any variable as predictor of players’ national–international selection. This may be due to the smaller sample size of this sports class, as well as the possibility that coaches consider other variables (not related to physical performance—such as perceptual skills, technical ability, or tactical knowledge) when selecting players. However, objectively measuring these technical and tactical skills presents a challenge, as coaches and scouts often rely on their “clinical eye” or expert knowledge [30]. Additionally, individuals with brain damage often develop other comorbidities beyond physical impairments, such as cognitive or sensory difficulties (e.g., intellectual, behavioral, visual, or auditory comorbidities) [31]. This may be a distinguishing factor in player selection, which has not been examined in the present study.

Regarding the match-related physical requirements, in which only FT2 players were included for data analysis, the results revealed that international players covered greater distances across all running intensities and demonstrated superior high-intensity efforts (maximal velocity, moderate- and high-intensity ball contacts). High-intensity running emerged as the strongest predictor of international status in FT2, confirming that high-intensity efforts are key differentiators in this sport class among national and international players. In mainstream football, it has been shown that players with higher competitive levels tend to cover greater total distances [32] and at higher intensities [33]. However, the literature presents conflicting findings on this matter, depending on the specific sample selected [34,35]. Specifically, in CP football, the only previous study analyzing differences in in-match physical responses between players of different competitive levels was conducted by Henríquez et al. (2024) [36]. Their findings indicated that higher-level players covered greater walking distances and during low-intensity running, whereas no significant differences were observed in high-intensity variables [36]. In the present study, differences between national and international players were observed in total distance covered, as well as in the distances covered at low, moderate, and high intensities, but not in the distance covered while walking. Our results may be more aligned with the literature on mainstream football [33,35]. However, other contextual variables, such as the competitive level of the opponent, match outcome, pacing strategies, and fatigue, appear to be influential factors in the physical responses observed during football matches [37], something to be considered in future research on this topic.

Furthermore, higher values of ball contacts (total, moderate- and high-intensity) were also found for international players. This could indicate greater involvement in the game within their teams compared to national players, although no significant differences were observed in the number of low-intensity ball contacts. To the best of the authors’ knowledge, there is no previous scientific literature to compare these findings, either in mainstream football or in CP football. However, the authors hypothesize that higher-level players tend to perform technical actions that require greater force when contacting the ball. Additionally, the greater functionality (lower impairment) of CP footballers selected for international competition could also explain these results. It should also be noted that the relatively high standard deviations observed in ball contact variables reflect the large inter-individual variability in this measure, with some players registering very few contacts relative to played time, while others accumulated higher counts; however, all values remained non-negative, as expected for this variable. Although no significant differences were found, international players also appeared to exhibit higher striking force, with a moderate effect size, which may align with the aforementioned arguments. This suggests that superior physical performance not only enhances movement efficiency but also contributes to greater technical involvement in match play. These results highlight the importance of integrating physical and technical assessments when identifying and selecting players for international competition. Further research is needed to determine the role of the quantity and force of ball striking in sport performance in CP football.

However, no significant differences between subgroups were found for moderate- and high-intensity accelerations and decelerations. Similarly, in mainstream football, acceleration and deceleration capacities do not appear to discriminate between playing levels [38]. Nevertheless, previous research in CP football has shown that these short-term high-intensity actions can differentiate between sport classes, particularly highlighting that players with mild impairments (FT8) perform significantly more moderate and high accelerations and decelerations than players with greater functional limitations (FT5/6, FT7) [5]. Moreover, accelerative and decelerative demands seem to be influenced more by the players’ level of impairment than by contextual factors such as team ranking or match outcome [36]. These findings suggest that while accelerations and decelerations may not serve as reliable indicators for distinguishing international performance levels in CP football, they remain relevant variables within the classification framework, especially to characterize the neuromuscular capabilities associated with different impairment profiles.

This study presents some limitations that should be considered. One limitation of the study is the small sample size in FT1 and FT3 players, which led to low statistical power in the Mann–Whitney U test comparisons for these groups (n < 5 per group), so these results should be interpreted with caution. However, it is important to highlight that this study included the entire population of Spanish CP football players competing at the national and international levels, making the findings highly representative of this sport’s competitive reality (i.e., Spain is top 10 world-ranked by the IFCPF and bronze medal in the last European Championships). To overcome this limitation, future studies should expand the sample through multi-center collaborations involving different national federations, which would increase the number of FT1 and FT3 players and improve the statistical robustness of the analyses. Additionally, while wearable GPS devices provided valuable insights into in-match physical responses, they did not account for tactical variables such as team formations, playing styles, or ball possession, which may also influence physical outputs. Future studies could address this by combining sensor-based data with notational analysis, game performance indicators, or advanced tracking tools (e.g., video-based tactical analysis, semi-automated match analysis systems) to integrate tactical and contextual dimensions. In addition, the study did not include specific measures of technical performance (e.g., pass accuracy, shooting efficiency) or tactical decision-making (e.g., response time), which may also play a relevant role in differentiating performance levels, particularly in players with higher functional capacity such as those in the FT3 class. Future studies should combine physical monitoring with technical and cognitive-tactical indicators to enhance the discriminatory power of the analyses. Importantly, the current study did not consider role division within teams (e.g., defenders, midfielders, attackers), which could partly explain the observed differences in running demands between national and international FT2 players. Therefore, our findings regarding high-speed running as a key differentiator may reflect, at least in part, tactical role allocation rather than purely physical capacity. However, we hypothesize that this potential effect might have been partially controlled, as the playing positions and roles of the participants were not fixed but rather varied across matches, with players from each sport class (including FT2) occupying different positions and tactical roles. This random allocation across roles may have mitigated, at least to some extent, the influence of positional bias on the results. Another methodological limitation concerns the placement of the OLIVER device on the back of the dominant leg, which, although useful for detecting ball contacts, may not fully capture actions performed with the non-dominant leg or reflect potential asymmetries in players with CP. Future research could explore bilateral or additional sensor placements to provide a more comprehensive assessment of asymmetry in CP football.

These findings have significant implications for talent identification and international selection in CP football players. Given the differences in sprinting, change of direction, and dribbling skills between national and international players, talent identification programs should incorporate assessments of these abilities, recognizing which of them play a more decisive role in the selection of international players in the different sport classes. This identification and selection process should continue to be complemented by the technical expertise of coaches and staff, as technical and tactical variables—not analyzed in the present study—may also play a crucial role in sport performance in CP football. Additionally, monitoring in-match physical responses through wearable technology can provide valuable insights into a player’s suitability for international competition. Players who cover greater distances (total and at various intensities), reach higher speeds, and register a greater number of moderate- and high-intensity ball contacts could be considered potential talents. When evaluated alongside the previously mentioned variables, these factors may contribute to the selection of players for an international-level competition.

## 5. Conclusions

This study provides new evidence on the differences in physical performance and in-match physical responses between national and international CP football players. The results confirm that international players exhibit superior performance in sprinting, change of direction, and dribbling skills, as well as greater high-intensity running capacity during matches, particularly among FT2 players. Different variables should be considered as primary factors in the talent identification and international selection processes across the various sport classes. Sprinting and change of direction ability appear to be more important in the FT1 sport class, whereas dribbling ability gains greater relevance for FT2 and FT3 players. For FT3 players, no single variable clearly predicts international selection, suggesting that factors other than physical performance may play a role in their selection process. Overall, this study provides valuable insights for coaches, scouts, and sports scientists involved in CP football, offering an evidence-based approach to enhancing talent identification and selection strategies in this team para-sport discipline.

## Figures and Tables

**Table 1:** National and international descriptive data for field tests.

Variable	FT1 National	FT1 International	FT2 National	FT2 International	FT3 National	FT3 International
Body weight (kg)	65.96 ± 10.33	69.08 ± 5.57	71.08 ± 8.82	67.00 ± 6.94	63.90 ± 7.72	67.33 ± 2.52
Height (m)	1.73 ± 0.08	1.82 ± 0.08	1.75 ± 0.06	1.77 ± 0.07	1.73 ± 0.12	1.73 ± 0.02
5 m (s)	1.46 ± 0.19	1.26 ± 0.09	1.28 ± 0.13	1.19 ± 0.09	1.24 ± 0.09	1.19 ± 0.05
10 m (s)	2.47 ± 0.34	2.11 ± 0.13	2.13 ± 0.17	2.00 ± 0.10	2.08 ± 0.10	1.98 ± 0.08
20 m (s)	4.29 ± 0.65	3.64 ± 0.23	3.62 ± 0.28	3.40 ± 0.15	3.51 ± 0.21	3.34 ± 0.16
30 m (s)	6.09 ± 0.99	4.76 ± 0.54	5.08 ± 0.41	4.74 ± 0.22	4.88 ± 0.32	4.64 ± 0.28
MAT (s)	7.42 ± 0.80	5.82 ± 0.94	6.57 ± 0.78	6.06 ± 0.60	6.59 ± 0.60	5.64 ± 0.22
Drib (s)	12.78 ± 2.70	9.83 ± 1.25	10.93 ± 1.65	8.93 ± 0.80	11.07 ± 2.40	8.28 ± 0.67
Drib-Ab (s)	5.49 ± 2.12	4.01 ± 1.58	4.35 ± 1.14	2.99 ± 0.59	4.48 ± 1.97	2.64 ± 0.53

MAT: modified agility T-test; Drib-Ab: dribbling ability.

**Table 2:** Multiple discriminant analysis data for each sport class.

	FT1			FT2			FT3		
	Wilks’ Lambda	F	*p*	Wilks’ Lambda	F	*p*	Wilks’ Lambda	F	*p*
5 m sprint	0.81	4.45 *	0.048	0.84	7.10 *	0.011	0.99	0.10	0.763
10 m sprint	0.81	4.43 *	0.049	0.81	8.79 *	0.005	0.93	0.55	0.481
15 m sprint	0.83	3.85	0.064	0.79	9.95 *	0.003	0.93	0.53	0.492
20 m sprint	0.83	4.02	0.059	0.79	10.29 *	0.003	0.96	0.28	0.613
30 m sprint	0.74	6.64 *	0.019	0.77	11.57 *	0.002	0.99	0.11	0.751
MAT	0.61	12.23 *^#^	0.002	0.82	8.26 *	0.007	0.67	3.48	0.105
Dribbling	0.80	4.70 *	0.043	0.70	16.05 *^#^	<0.001	0.77	2.12	0.189
Drib-Ab	0.92	1.69	0.209	0.72	14.61 *	<0.001	0.83	1.45	0.267

MAT: modified agility T-test; Drib-Ab: dribbling ability. * *p* < 0.05; ^#^ Main predictable variable for the international selection.

**Table 3:** Mann–Whitney U test for in-match physical requirements comparison between national and international FT2 players.

	National	International	U	*p*	ES (95% CI)
Average playing time (min)	61.53 ± 18.55	67.93 ± 15.25	272.50	0.362	0.16 (−0.17; 0.46)
Maximal velocity (k·h^−1^)	23.38 ± 2.75	25.19 ± 2.19	187.00	0.013	0.42 (0.12; 0.65)
Total distance (m·min^−1^)	57.05 ± 18.90	68.70 ± 13.92	209.00	0.038	0.35 (0.04; 0.61)
LI ball contacts (n·min^−1^)	0.07 ± 0.09	0.07 ± 0.04	236.50	0.117	0.27 (−0.06; 0.54)
MI ball contacts (n)	0.09 ± 0.07	0.14 ± 0.07	205.00	0.032	0.37 (0.05; 0.62)
HI ball contacts (n)	0.09 ± 0.08	0.14 ± 0.08	178.50	0.009	0.45 (0.15; 0.67)
Ball contacts (n)	0.25 ± 0.22	0.35 ± 0.18	195.50	0.021	0.40 (0.08; 0.64)
Striking force (k·h^−1^)	45.51 ± 10.55	50.92 ± 6.51	222.00	0.067	0.31 (−0.01; 0.58)
LI running (m·min^−1^)	20.26 ± 7.79	25.95 ± 6.00	169.00	0.004	0.48 (0.18; 0.69)
MI running (m·min^−1^)	5.97 ± 3.48	8.85 ± 3.00	176.50	0.008	0.45 (0.16; 0.68)
HI running (m·min^−1^)	0.32 ± 0.37	0.63 ± 0.66	150.00	0.002	0.54 (0.26; 0.73)
Walking (m·min^−1^)	28.64 ± 9.44	30.60 ± 6.49	307.00	0.780	0.05 (−0.28; 0.37)
MI accelerations (m·min^−1^)	3.18 ± 1.71	3.80 ± 1.07	217.50	0.056	0.33 (0.01; 0.59)
HI accelerations (m·min^−1^)	1.24 ± 0.81	1.63 ± 0.77	220.00	0.061	0.32 (−0.01; 0.58)
MI decelerations (m·min^−1^)	3.42 ± 1.55	3.95 ± 1.08	253.50	0.209	0.22 (−0.11; 0.50)
HI decelerations (m·min^−1^)	1.66 ± 0.98	2.01 ± 0.81	227.50	0.084	0.30 (−0.03; 0.56)

LI: low-intensity; MI: moderate-intensity; HI: high-intensity; ES: effect size; CI: confidence interval.

## Data Availability

The data supporting the findings of this study are not publicly available due to privacy and ethical restrictions. Interested researchers may contact the corresponding author for further information, where appropriate and in compliance with applicable regulations.
